# Absorption of α-tocopheryl acetate is limited in mink kits (*Mustela vison*) during weaning

**DOI:** 10.1038/s41598-020-80902-0

**Published:** 2021-01-29

**Authors:** Saman Lashkari, Tove N. Clausen, Leslie Foldager, Søren K. Jensen

**Affiliations:** 1 Department of Animal Science, Aarhus University, Blichers Allé 20, 8830 Tjele, Denmark; 2 Copenhagen Fur Research Centre, Herningvej 112 C, 7500 Holstebro, Denmark; 3 Bioinformatics Research Centre, Aarhus University, C.F. Møllers Allé 8, 8000 Aarhus C, Denmark

**Keywords:** Biochemistry, Biological techniques, Chemical biology

## Abstract

Bioavailability of α-tocopherol varies with source, dose and duration of supplementation. The effect of source and dose of α-tocopherol on response of α-tocopherol stereoisomers in plasma and tissues of mink kits during the weaning period was studied. Twelve mink kits were euthanised in CO_2_ at the beginning of the experiment, and 156 mink kits (12 replicates per treatment group) were randomly assigned to thirteen treatment groups: no added α-tocopherol in the feed (0 dose) or four different doses (50, 75, 100 and 150 mg/kg of diet) of RRR-α-tocopherol (ALC), RRR-α-tocopheryl acetate (ACT) or *all-rac*-α-tocopheryl acetate (SYN). Six mink kits per treatment group were euthanised 3 weeks after initiation of the experiment, and the remaining six were euthanised 6 weeks after initiation of the experiment. The RRR-α-tocopherol content in plasma, liver, heart and lungs was affected by interaction between source and dose (*P* < 0.01 for all). The highest RRR-α-tocopherol content in plasma (13.6 µg/ml; LS-means for source across dose and week), liver (13.6 µg/mg), heart (7.6 µg/mg) and lungs (9.8 µg/mg) was observed in mink kits fed ALC. The RRR-α-tocopherol content in plasma and tissues depended on source and dose interaction and increased linearly with supplementation. In conclusion, the interaction between source and dose reveals a limitation in hydrolysis of ester bond in α-tocopheryl acetate in mink kits around weaning as the likely causative explanation for the higher response of ALC at the highest doses. Thus, considerable attention has to be paid to the source of α-tocopherol during weaning of mink kits fed a high dose of α-tocopherol.

## Introduction

Sufficient supply of α-tocopherol is critical for appropriate growth and an effective immune system^1^ and antioxidant function^2^. Mink diets are characterized by a high protein content and fat content, often of marine origin and high in polyunsaturated fatty acids. In order to cope with such a high content of polyunsaturated fatty acids, mink need a considerable amount of antioxidants like vitamin E^3^.

Synthetic manufactured racemic α-tocopheryl acetate (*all-rac*-α-tocopheryl acetate) is the typical and most important source of α-tocopherol supplement in animal and human nutrition. Synthetic α-tocopherol consists of eight different isomeric configurations, including four with 2R configuration (RSS, RRS, RSR, RRR) and four with 2S configuration (SRR, SSR, SRS, SSS). The RRR stereoisomer is the only form of α-tocopherol occurring in nature and is also available as feed additive. In most commercial vitamin premix, RRR-α-tocopherol and *all-rac*-α-tocopherol are acetylated in order to stabilize the functional phenol group during storage. The acetylated form of α-tocopherol must be hydrolysed in the intestinal tract prior to absorption. Hydrolysis of the ester bond in α-tocopheryl acetate is facilitated by pancreatic carboxyl ester hydrolase and may be the limiting factor in the absorption of α-tocopherol in young farm animals, such as newly weaned piglets and calves^2^, and in individuals with impaired lipid absorption capacity^4^. Hydrolysis of the ester bond is not considered to be a limiting factor for absorption of α-tocopherol during weaning in mink in diets containing 65 to 70 mg/kg of α-tocopheryl acetate^3^. However, it remains unclear whether the hydrolysis capacity of α-tocopheryl acetate becomes a limiting factor at higher levels of vitamin E doses in the diets.

The official biopotency factors of 1.00 for *all-rac*-α-tocopheryl acetate and 1.36 for RRR-α-tocopheryl acetate based on the rat resorption-gestation test^5^ have been challenged. For instance, the relative bioavailability is not constant^6^ but depends on duration of administration, dose of α-tocopherol and target tissue^6,7^. Hymøller et al.^7^ reported that increasing dosage of *all-rac*-α-tocopheryl acetate decreased plasma proportion of RRR-α-tocopherol. Despite the comparable absorption, differential retention of α-tocopherol stereoisomers results in different bioavailability with the duration of dosing. Preferential retention of the 2R forms and elimination of the 2S forms after every dose result in a progressively greater proportion of retained α-tocopherol being 2R forms^8,9^. In addition, human studies demonstrated that bioavailability of α-tocopherol stereoisomers varies with dosage^6^. The purpose of the present experiment was to investigate the effect of stereoisomeric configuration, free or acetylated form of α-tocopherol at different doses of supplementation on plasma and tissue responses as well as bioavailability of RRR-α-tocopherol in mink kits during the weaning period. Weaning of mink kits occured gradually from an age of 4 weeks (beginning of experiment) to 7 weeks of age (corresponding to 3 weeks of administration of α-tocopherol). Last sampling was at 10 weeks of age (6 weeks after administration of α-tocopherol).

## Results

Analysis of the α-tocopherol content and distribution of stereoisomers in experimental diets for the different treatment groups is shown in Table 1 and is in line with the planned content of α-tocopherol in the supplemented treatment groups. At the beginning of the experiment, the level of α-tocopherol was 25.1 ± 5.5 (means ± SD, µg/ml) in plasma, 107 ± 5.0 (µg/g) in liver, 1.3 ± 0.4 (µg/g) in heart and 13.3 ± 3.4 (µg/g) in lungs. Live body weight was 194 ± 24, 534 ± 102 and 1038 ± 174 g (means across treatment groups ± SD) in week 0, 3 and 6, respectively, which was in the normal range.

**Table 1:** α-Tocopherol content and distribution of stereoisomers in the different experimental diets (Means ± SD).

	Control	Source and dose of α-tocopherol											
		RRR-α-tocopherol (ALC, mg/kg of diet)				RRR-α-tocopheryl acetate (ACT, mg/kg of diet)				*All-rac*-α-tocopheryl acetate (SYN, mg/kg of diet)			
	0	50	75	100	150	50	75	100	150	50	75	100	150
α-Tocopherol	10.3 ± 0.6	58.5 ± 0.8	69.6 ± 1.3	92.8 ± 0.2	153.5 ± 0.6	52.2 ± 0.1	78.8 ± 1.5	102.6 ± 3.2	145.9 ± 0.5	59.5 ± 0.1	78.8 ± 1.0	96.1 ± 1.4	156.5 ± 0.9
Composition of α-tocopherol stereoisomers (%)													
RRR	100	91.7	94.4	94.4	95.3	86.3	86.9	87.1	85.9	28.8	21.5	21.4	16.3
RRS	ND^a^	2.3	1.5	1.6	1.3	1.7	1.5	1.2	1.8	11.2	12.2	11.0	11.8
RSS	ND	3.5	1.8	1.9	1.9	1.5	1.9	1.5	2.9	11.9	12.4	11.1	12.0
RSR	ND	1.0	1.1	1.2	0.7	2.9	1.7	2.1	1.8	10.6	11.0	11.2	11.6
2S	ND	1.4	1.2	0.9	0.7	7.6	8.0	8.1	7.6	37.5	43.0	45.4	48.3

^a^ND, Not detected.

2S = Summation of 2S configuration of α-tocopherol stereoisomers.

### Response to different sources of α-tocopherol

**Figure 1:**
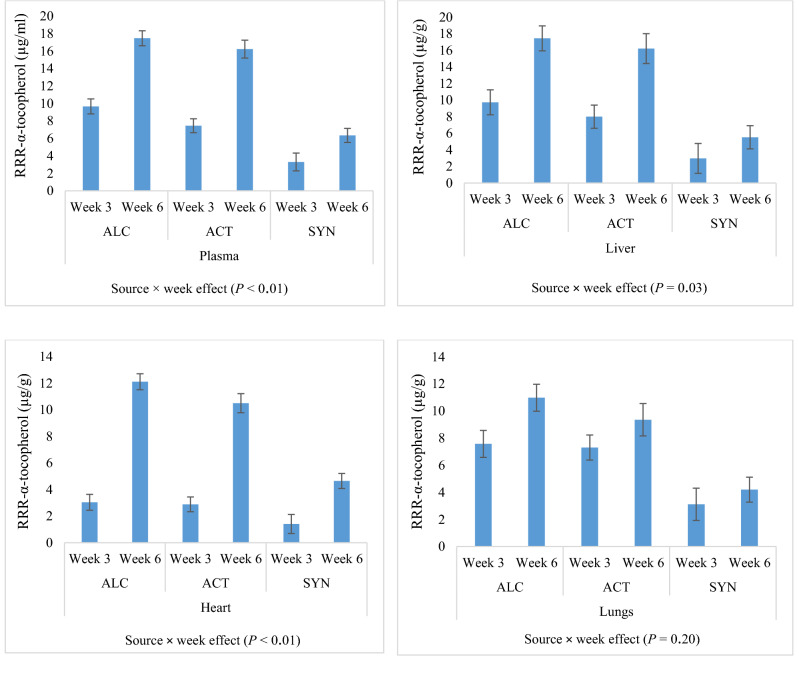
RRR-α-tocopherol of plasma, liver, heart and lungs 3 and 6 weeks after supplementation of RRR-α-tocopherol (ALC), RRR-α-tocopheryl acetate (ACT) and *all-rac*-α-tocopheryl acetate (SYN). Presented values are LS-means across dose ± SEM.

Fat-soluble vitamins and α-tocopherol stereoisomers of different treatments in plasma, liver, heart and lungs are presented in Tables 2, 3. The plasma α-tocopherol content was 14.0 (LS-means for source across dose and week; values not shown in Tables; µg/ml) in ALC, 12.4 (µg/ml) in ACT and 10.2 (µg/ml) in SYN and affected by source of α-tocopherol (*P* < 0.01). Plasma RRR-α-tocopherol was affected by interaction between source of α-tocopherol and week (*P* < 0.01) and interaction between source and dose of α-tocopherol (*P* < 0.01). The plasma RRR-α-tocopherol content was highest for ALC (13.6 µg/ml), intermediate for ACT (11.8 µg/ml) and lowest for SYN (4.8 µg/ml). In addition, the results showed higher plasma RRR-α-tocopherol in ALC than both ACT and SYN in 75, 100 and 150 mg/kg of diets in both weeks 3 and 6. The highest plasma content of synthetic 2R-α-tocopherol (4.7 µg/ml) and 2S-α-tocopherol (0.6, µg/ml) were observed in SYN, and significant interaction was observed between source and dose of α-tocopherol (*P* < 0.01 for both synthetic 2R and 2S-α-tocopherol). The plasma RRR-α-tocopherol content was influenced by interaction between week and source of α-tocopherol (Fig. 1), and irrespective of the α-tocopherol dose, a higher plasma α-tocopherol content was observed 6 weeks after supplementation compared to 3 weeks after supplementation. The plasma content of γ-tocopherol was affected by interaction between source of α-tocopherol and dose (*P* < 0.01). The retinol content in plasma was affected by week (*P* < 0.01) and was highest at 75 and 100 mg/kg in ALC. The plasma content of 25-OH-D3 was 42.7, 50.9 and 52.9 (µg/ml) in ALC, ACT and SYN, respectively, and affected by interaction between source and dose of α-tocopherol (*P* < 0.01).

**Table 2:** Fat-soluble vitamins and α-tocopherol stereoisomers of plasma (µg/ml) of mink kits fed different sources (ALC, ACT and SYN) and doses of α-tocopherol (0, 50, 75, 100 and 150 mg/kg of diet).

	Source and dose of α-tocopherol															SEM	*P-*values						
	RRR-α-tocopherol (ALC)					RRR-α-tocopheryl acetate (ACT)					*All-rac*-α-tocopheryl acetate (SYN)												
	0	50	75	100	150	0	50	75	100	150	0	50	75	100	150		S	D	W	S × D	S × W	D × W	S × D × W
**Plasma**																							
α-Tocopherol	3.03	11.91	15.12	18.03	21.81	5.13	12.4	12.7	13.3	18.6	4.12	8.9	10.2	11.7	15.9	1.2	< 0.01	< 0.01	< 0.01	0.30	0.27	0.02	0.93
RRR	2.66	11.20	14.68	17.61	21.69	4.53	11.8	12.1	12.7	18.0	3.66	4.30	4.76	4.99	6.43	1.12	< 0.01	< 0.01	< 0.01	< 0.01	< 0.01	0.02	0.70
RRS	0.17	0.49	0.32	0.28	0.11	0.26	0.30	0.21	0.24	0.22	0.21	1.87	1.83	2.22	3.35	0.19	< 0.01	< 0.01	< 0.01	< 0.01	< 0.01	0.59	0.55
RSS	0.07	0.10	0.06	0.05	0.01	0.12	0.14	0.14	0.12	0.10	0.09	1.09	1.45	1.78	2.46	0.09	< 0.01	< 0.01	< 0.01	< 0.01	< 0.01	< 0.41	0.19
RSR	0.08	0.10	0.05	0.04	0.01	0.15	0.17	0.11	0.13	0.09	0.13	1.19	1.53	1.88	2.58	0.09	< 0.01	< 0.01	< 0.01	< 0.01	< 0.01	< 0.20	0.09
Synthetic 2R	0.33	0.68	0.42	0.37	0.12	0.54	0.60	0.46	0.48	0.40	0.43	4.14	4.78	5.87	8.39	0.31	< 0.01	< 0.01	< 0.01	< 0.01	< 0.01	0. 53	0.29
2S	0.04	0.05	0.08	0.16	0.08	0.06	0.07	0.07	0.09	0.13	0.02	0.49	0.61	0.79	1.12	0.05	< 0.01	< 0.01	< 0.01	< 0.01	< 0.01	0.99	0.71
2R	2.90	11.9	15.1	18.0	21.8	5.07	12.4	12.6	13.2	18.4	4.10	8.4	9.6	10.9	14.8	1.22	< 0.01	< 0.01	< 0.01	0.19	0.19	0.02	0.92
γ-Tocopherol	0.08	0.08	0.07	0.04	0.05	0.14	0.18	0.06	0.05	0.06	0.09	0.08	0.06	0.08	0.06	0.01	0.06	< 0.01	0.80	< 0.01	0.15	0.02	< 0.01
Retinol	1.81	2.18	2.79	3.58	2.71	1.95	3.20	3.38	2.72	2.35	1.76	2.48	2.67	2.90	2.24	0.29	0.20	< 0.01	< 0.01	0.12	0.30	0.28	0.57
25-OH-D3	51.3	40.9	42.4	33.7	45.4	53.1	49.9	54.4	45.8	51.5	32.9	55.6	62.5	57.9	55.6	3.8	< 0.01	0.10	< 0.01	0.01	0.53	0.15	0.69

Synthetic 2R = Summation of synthetic 2R configuration of α-tocopherol stereoisomers (ƩRRS, RSS, RSR), 2S = Summation of S configuration of α-tocopherol stereoisomers, 2R = Summation of R configuration of α-tocopherol stereoisomers, S = Effect of source, D = Effect of dose, W = Effect of weeks, S × D = Interaction between source and dose, S × W = Interaction between source and weeks, D × W = Interaction between dose and weeks, S × D × W = Interaction between source, dose and weeks. Presented values in Table are LS-means of weeks 3 and 6. SEM for the dose 0 in ALC, ACT and SYN is slightly higher. Presented *P*-values are from type-2 tests.

**Table 3:** Fat-soluble vitamins and α-tocopherol stereoisomers of liver (µg/g), heart (µg/g) and lungs (µg/g) of mink kits fed different sources (ALC, ACT and SYN) and doses of α-tocopherol (0, 50, 75, 100 and 150 mg/kg of diet).

	Source and dose of α-tocopherol															SEM	*P-*values						
	RRR-α-tocopherol (ALC)					RRR-α-tocopheryl acetate (ACT)					*All-rac*-α-tocopheryl acetate (SYN)												
	0	50	75	100	150	0	50	75	100	150	0	50	75	100	150		S	D	W	S × D	S × W	D × W	S × D × W
**Liver**																							
α-Tocopherol	4.15	11.3	14.6	20.0	26.0	3.91	10.7	12.8	13.4	29.1	3.25	12.8	13.9	20.2	29.7	2.4	0.36	< 0.01	< 0.01	0.78	0.78	0.23	0.99
RRR	2.99	9.71	13.05	18.17	24.06	3.01	9.12	11.2	11.55	25.6	2.44	4.13	3.67	4.52	6.46	1.95	< 0.01	< 0.01	< 0.01	< 0.01	0.03	0.24	0.98
RRS	0.38	1.03	1.02	1.21	1.21	0.37	0.37	0.34	0.40	0.53	0.28	1.47	1.56	2.16	4.29	0.23	< 0.01	< 0.01	0.02	< 0.01	< 0.01	0.86	0.90
RSS	0.21	0.25	0.28	0.30	0.44	0.22	0.29	0.27	0.30	0.39	0.17	1.37	1.62	2.25	2.78	0.16	< 0.01	< 0.01	< 0.01	< 0.01	< 0.01	< 0.01	< 0.01
RSR	0.20	0.13	0.10	0.11	0.10	0.10	0.19	0.18	0.22	0.32	0.10	1.28	1.47	2.13	3.51	0.14	< 0.01	< 0.01	< 0.01	< 0.01	< 0.01	0.06	< 0.01
Synthetic 2R	0.79	1.40	1.39	1.62	1.75	0.68	0.85	0.79	0.92	1.42	0.87	4.12	4.65	6.54	10.5	0.40	< 0.01	< 0.01	< 0.01	< 0.01	< 0.01	0.15	0.03
2S	0.39	0.21	0.17	0.21	0.21	0.21	0.78	0.82	0.95	2.22	0.26	4.55	5.56	9.16	12.65	0.65	< 0.01	< 0.01	0.99	< 0.01	0.97	0.95	0.99
2R	3.77	11.11	14.45	19.79	25.82	3.77	9.98	12.0	12.47	26.9	2.99	8.25	8.32	11.1	17.4	2.13	< 0.01	< 0.01	< 0.01	0.39	0.64	0.14	0.99
Retinol	262	271	313	268	301	254	289	275	284	260	270	304	296	350	353	18.70	< 0.01	0.24	< 0.01	0.15	0.41	0.71	0.64
**Heart**																							
α-Tocopherol	1.67	6.10	8.40	11.01	13.27	2.98	6.24	6.21	8.80	11.8	2.30	5.57	6.40	7.84	9.71	0.97	< 0.01	< 0.01	< 0.01	0.61	0.02	< 0.01	0.51
RRR	1.43	5.50	7.90	10.39	12.63	2.49	5.71	5.80	8.19	11.2	2.15	2.88	2.98	3.30	3.85	0.80	< 0.01	< 0.01	< 0.01	< 0.01	< 0.01	< 0.01	0.08
RRS	0.11	0.38	0.34	0.42	0.41	0.22	0.24	0.18	0.26	0.25	0.08	0.96	1.18	1.54	2.15	0.11	< 0.01	< 0.01	< 0.01	< 0.01	< 0.01	0.46	0.27
RSS	0.05	0.14	0.10	0.14	0.16	0.13	0.15	0.12	0.18	0.16	0.04	0.74	0.93	1.21	1.52	0.08	< 0.01	< 0.01	< 0.01	< 0.01	< 0.01	0.68	0.72
RSR	0.07	0.05	0.05	0.04	0.04	0.10	0.10	0.07	0.12	0.10	0.04	0.71	0.90	1.23	1.54	0.08	< 0.01	< 0.01	< 0.01	< 0.01	< 0.01	0.66	0.62
Synthetic 2R	0.22	0.57	0.48	0.60	0.60	0.46	0.49	0.37	0.57	0.50	0.17	2.40	3.02	3.99	5.22	0.27	< 0.01	< 0.01	< 0.01	< 0.01	< 0.01	0.60	0.56
2S	0.01	0.04	0.01	0.02	0.04	0.02	0.04	0.04	0.05	0.08	0.005	0.29	0.41	0.55	0.64	0.04	< 0.01	< 0.01	< 0.01	< 0.01	< 0.01	0.70	0.94
2R	1.66	6.07	8.39	10.99	13.23	2.96	6.20	6.17	8.75	11.7	2.30	5.28	5.99	7.29	9.07	0.94	< 0.01	< 0.01	< 0.01	0.44	< 0.01	0.01	0.48
γ-Tocopherol	0.50	0.31	0.41	0.64	0.48	0.34	0.62	0.63	0.53	0.37	0.11	0.50	0.54	0.98	0.26	0.11	0.80	0.02	< 0.01	0.07	0.30	0.67	0.49
**Lungs**																							
α-Tocopherol	2.95	8.32	9.89	16.20	11.66	3.88	8.15	8.06	8.65	15.5	3.44	7.46	9.50	8.39	9.64	1.46	0.05	< 0.01	< 0.01	< 0.01	0.52	0.55	0.81
RRR	2.41	7.51	9.47	15.70	11.29	3.36	7.48	7.70	8.19	14.9	2.94	3.77	4.26	3.45	3.84	1.31	< 0.01	< 0.01	< 0.01	< 0.01	0.20	0.47	0.65
RRS	0.26	0.54	0.30	0.38	0.26	0.23	0.30	0.12	0.18	0.28	0.26	1.24	1.75	1.57	1.93	0.13	< 0.01	< 0.01	0.53	< 0.01	< 0.01	0.40	0.45
RSS	0.07	0.13	0.08	0.05	0.06	0.04	0.15	0.09	0.10	0.10	0.07	0.92	1.38	1.25	1.34	0.10	< 0.01	< 0.01	0.83	< 0.01	0.02	0.99	0.99
RSR	0.12	0.07	0.03	0.05	0.03	0.12	0.12	0.08	0.09	0.13	0.10	0.94	1.38	1.33	1.60	0.10	< 0.01	< 0.01	0.44	< 0.01	0.02	0.96	0.99
Synthetic 2R	0.46	0.75	0.41	0.47	0.34	0.39	0.57	0.29	0.37	0.51	0.45	3.11	4.51	4.15	4.86	0.30	< 0.01	< 0.01	0.96	< 0.01	< 0.01	0.86	0.98
2S	0.08	0.07	0.01	0.02	0.02	0.08	0.09	0.07	0.08	0.15	0.06	0.58	0.73	0.78	0.93	0.06	< 0.01	< 0.01	< 0.01	< 0.01	0.06	0.27	0.93
2R	3.78	8.25	9.88	16.18	11.64	2.96	8.06	7.99	8.56	15.4	3.39	6.88	8.77	7.61	8.71	1.43	< 0.01	< 0.01	< 0.01	< 0.01	0.55	0.56	0.79

Synthetic 2R = Summation of synthetic 2R configuration of α-tocopherol stereoisomers (ƩRRS, RSS, RSR), 2S = Summation of S configuration of α-tocopherol stereoisomers, 2R = Summation of R configuration of α-tocopherol stereoisomers, S = Effect of source, D = Effect of dose, W = Effect of weeks, S × D = Interaction between source and dose, S × W = Interaction between source and weeks, D × W = Interaction between dose and weeks, S × D × W = Interaction between source, dose and weeks. Presented values in Table are LS-means of weeks 3 and 6. SEM for the dose 0 in ALC, ACT and SYN is slightly higher. Presented *P*-values are from type-2 tests.

The liver RRR-α-tocopherol content was affected by interaction between source and dose of α-tocopherol (*P* < 0.01) and interaction between source of α-tocopherol and week (*P* = 0.03). The RRR-α-tocopherol content of liver was 13.6, 12.1 and 4.2 (µg/g) in ALC, ACT and SYN, respectively. In liver, the highest amount of synthetic 2R-α-tocopherol (5.3 µg/g) and 2S-α-tocopherol (6.4 µg/g) was observed in SYN, and significant interaction was observed between the source and dose of α-tocopherol (*P* < 0.01 for both synthetic 2R and 2S-α-tocopherol). Irrespective of the α-tocopherol dose, the liver RRR-α-tocopherol content was higher 6 weeks after supplementation than 3 weeks after supplementation, and the interaction between week and source of α-tocopherol was significant (Fig. 1, *P* = 0.03). The retinol content in the liver was highest in SYN, intermediate in ALC and lowest in ACT (*P* < 0.01).

The α-tocopherol content in heart was affected by interaction between source of α-tocopherol and week (*P* < 0.01), and it was 8.1, 7.2 and 6.4 (µg/g) in ALC, ACT and SYN, respectively. The RRR-α-tocopherol content of heart was affected by interaction between source of α-tocopherol and week (*P* < 0.01) and interaction between α-tocopherol source and dose (*P* < 0.01). The RRR-α-tocopherol content of heart was 7.6 (µg/g) in ALC, 6.7 (µg/g) in ACT and 3.0 (µg/g) in SYN. The highest content of synthetic 2R-α-tocopherol (2.9 µg/g) and 2S-α-tocopherol (0.38 µg/g) in heart was observed in SYN, and significant interaction was observed between α-tocopherol source and dose of α-tocopherol (*P* < 0.01 for both synthetic 2R and 2S α-tocopherol). The RRR-α-tocopherol of heart was influenced by interaction of source of α-tocopherol and week (*P* < 0.01), and the heart RRR-α-tocopherol content increased from week 3 to 6 of the experiment (Fig. 1).

The α-tocopherol content of lungs was affected by interaction between source and dose of α-tocopherol (*P* < 0.01), and it was 9.8 (µg/g) in ALC, 8.8 (µg/g) in ACT and 7.6 (µg/g) in SYN. Similar interaction between source and dose of α-tocopherol was observed for the RRR-α-tocopherol content, and it was 9.2, 8.3 and 3.6 (µg/g) in ALC, ACT and SYN, respectively. Similar to plasma, liver and heart, the highest amount of synthetic 2R-α-tocopherol (3.4 µg/g) and 2S-α-tocopherol (0.6 µg/g) in lungs was observed in SYN, and significant interaction was observed between source and dose of α-tocopherol (*P* < 0.01 for both synthetic 2R and 2S α-tocopherol). Although the content of RRR-α-tocopherol in lungs was not influenced by the interaction between source of α-tocopherol and week (*P* = 0.20), the RRR-α-tocopherol content of lungs was numerically higher 6 weeks after supplementation than 3 weeks after supplementation (Fig. 1).

### Effect of dose and duration of α-tocopherol

**Figure 2:**
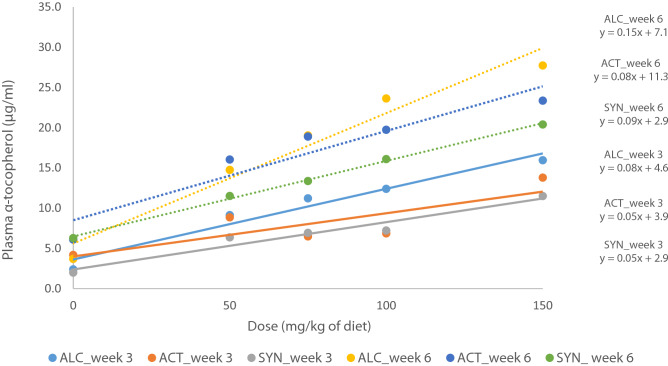
Linear correlation between the plasma α-tocopherol and dose of supplementation in mink kits fed diets supplemented with RRR-α-tocopherol after 6 weeks (ALC_week 6) and 3 weeks (ALC_week 3), RRR-α-tocopheryl acetate after 6 weeks (ACT_week 6) and 3 weeks (ACT_week 3) and *all-rac*-α-tocopheryl acetate after 6 weeks (SYN_week 6) and 3 weeks (SYN_week 3). Solid line and dashed line represent the LS-means for weeks 3 and 6, respectively.

Regression analysis showed a linear response between plasma α-tocopherol and dose of supplementation (Fig. 2) in both weeks 3 and 6. The interaction between week and source of α-tocopherol (*P* = 0.04, *P*-value from regression analysis and not presented in Tables) showed that the differences in intercept of plasma α-tocopherol between week 3 and week 6 depended on the source of α-tocopherol. The results demonstrated that the plasma α-tocopherol content was 11.2 and 6.5 µg/ml in mink kits fed 75 mg/kg of ALC and ACT in week 3, respectively, and mink kits fed 75 mg/kg of ALC had a higher plasma α-tocopherol content than mink kits fed ACT (+ 73%). Similarly, mink kits fed 100 mg/kg of ALC after 3 weeks of supplementation showed a higher plasma α-tocopherol content than those fed ACT (+ 80%). The plasma α-tocopherol content was 23.6 and 19.7 µg/ml in mink kits fed 100 mg/kg of ALC and ACT in week 6, respectively, and mink kits fed 100 mg/kg of ALC had a higher plasma α-tocopherol content compared to ACT in week 6 (+ 19%). A similar difference was observed in 150 mg/kg, and mink kits fed ALC had a higher plasma α-tocopherol content than the ACT (+ 18%).

**Figure 3:**
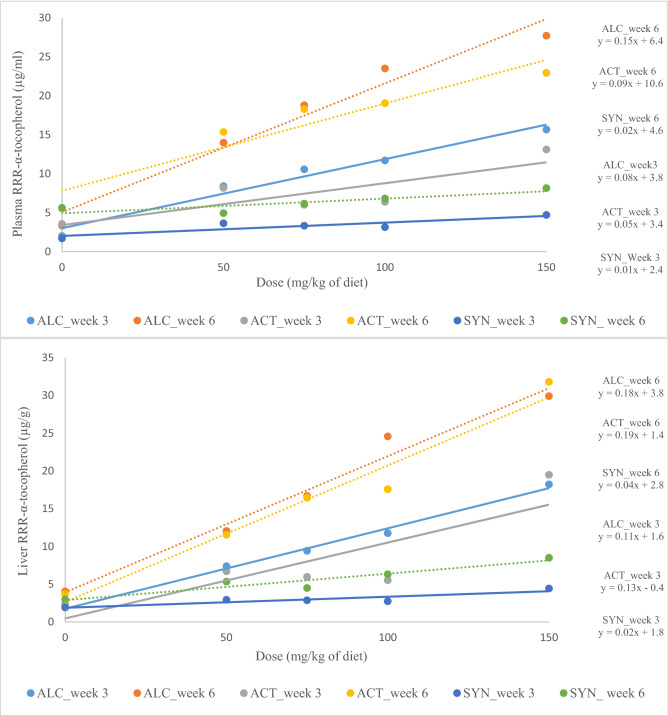

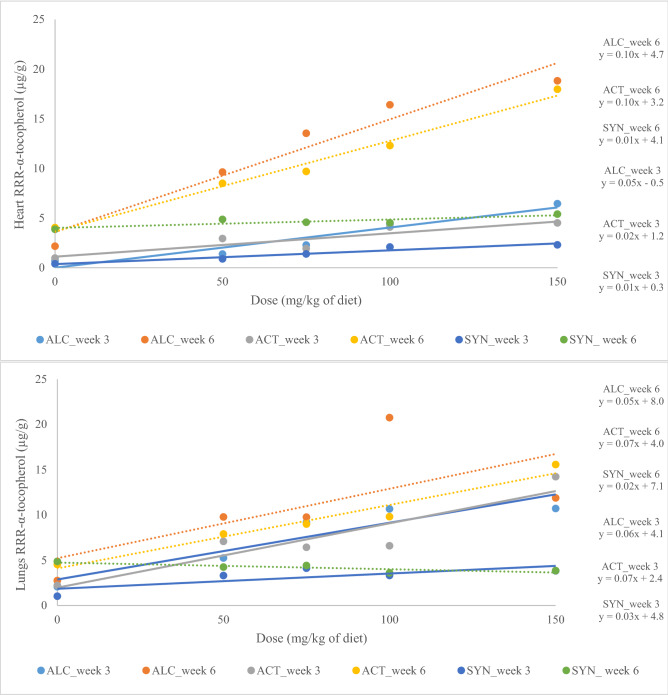
Linear correlation in plasma, liver, heart and lungs between the RRR-α-tocopherol and dose of supplementation in mink kits fed diets supplemented with RRR-α-tocopherol after 6 weeks (ALC_week 6) and 3 weeks (ALC_week 3), RRR-α-tocopheryl acetate after 6 weeks (ACT_week 6) and 3 weeks (ACT_week 3) and *all-rac*-α-tocopheryl acetate after 6 weeks (SYN_week 6) and 3 weeks (SYN_week 3). Solid line and dashed line represent the LS-means for weeks 3 and 6, respectively.

Regression analysis showed a linear response between RRR-α-tocopherol and dose of supplementation (Fig. 3) in plasma, liver, heart and lungs. The interaction between week and dose of supplementation (*P* = 0.01, *P*-value from regression analysis and not presented in Tables) showed that the response of RRR-α-tocopherol in plasma and tissues to different doses was different in weeks 3 and 6, and regardless of source of supplementation, RRR-α-tocopherol of plasma and tissues was higher in week 6 after supplementation than week 3 after supplementation. The RRR-α-tocopherol content in plasma, liver, heart and lungs increased more than 5, 8, 6 and fourfold after supplementation in mink kits fed 150 mg/kg of ALC compared to 0 mg, respectively. Similarly, the RRR-α-tocopherol in plasma, liver, heart and lungs increased more than 4, 9, 2 and fourfold in mink kits fed 150 mg/kg of ACT compared to 0 mg, respectively. The RRR-α-tocopherol in plasma and tissues increased less than twofold in SYN, which was lower than the response observed in ALC and ACT.

### Relative bioavailability of RRR-α-tocopherol in *all-rac*-α-tocopheryl acetate

**Figure 4:**
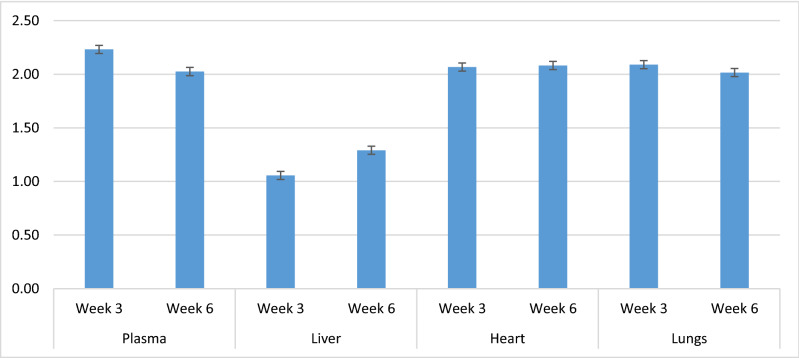
Relative bioavailability of RRR-α-tocopherol in plasma, liver, heart and lungs of mink fed 50, 75, 100 mg/kg of *all-rac*-α-tocopheryl acetate (SYN). Presented values are LS-means across dose ± SEM. The relative bioavailability of RRR-tocopherol in plasma and tissues was calculated as percentage of a given RRR-tocopherol in plasma and tissues divided by percentage of the RRR-tocopherol in the diet.

Figure 4 presents the relative bioavailability of RRR-α-tocopherol for 50, 75, 100 and 150 mg/kg of SYN in plasma and different tissues. Relative bioavailability of RRR-α-tocopherol in plasma and different tissues was influenced by the effect of tissue (*P* < 0.01), dose (*P* < 0.01), interaction between tissue and dose (*P* = 0.002) and interaction between tissue and week (*P* < 0.01), and the relative bioavailability of RRR-α-tocopherol in liver (1.17) was lower than plasma (2.1), heart (2.1) and lungs (2.1; LS-means across the dose and week, and values not shown in Tables).

## Discussion

Discrepancy between the α-tocopherol stereoisomer proportion in SYN diets and the theoretical proportion in *all-rac*-α-tocopheryl acetate (Table 1) reflects the contribution of RRR-α-tocopherol originated from basal diet. The same scenario is correct for synthetic stereoisomers seen in ALC and ACT due to using slaughter offal from poultry and farmed fish in basal diets originated from the *all-rac*-α-tocopheryl acetate fed to these animals.

The α-tocopherol status of the nursing mink kits was high at the beginning of the experiment (week 0) when mink kits only received vitamin E from milk. We have previously shown that mink milk contains around 7 mg of α-tocopherol per kg of milk, dominated by 2R stereosimers^10^, corresponding to less than 0.5 mg daily α-tocopherol intake from milk at 28 days of age^11^. From 28 days of age, the mink kits start to eat solid feed, and over the next 3 weeks the mink kits gradually changed to the solid diet. After a few days when the mink kits reached a daily intake of 10 g of solid feed, the α-tocopherol intake was already greater than 0.5 mg in the 50-mg groups. However, the α-tocopherol content of plasma, liver, heart and lungs was reduced to 11.3, 62.7, 2.9 and 22.5% of week 0 in the control group, respectively. Similarly, 6 weeks after starting the experiment, the α-tocopherol content of plasma, liver and lungs decreased to 21.3, 4.1 and 20.8% of week 0 in the control group. It is still unclear why this reduction occurs, most likely caused by a combination of poor absorption and physiological changes, which were observed in mammal species during weaning, i.e. piglet^12^, mink^7^, rat and human^13^. α-Tocopherol seems to be utilised very efficiently as long as the mink kits are milk-fed; however, during weaning, it is a great challenge to keep the α-tocopherol level high, which shows that special attention needs to be considered to the supply of α-tocopherol around and after weaning in mink kits. These findings are in agreement with previous study on mink^14^.

According to different levels of α-tocopherol in plasma and different tissues, the different sources of α-tocopherol fed to mink kits are not absorbed with equal efficiency, and it implies that the bioavailability of α-tocopherol is dependent on the source of α-tocopherol and duration of administration. Similar to Hymøller, et al.^3^, the present study also showed an interaction between the dose of α-tocopherol and plasma 25-OH-D_3_.

The higher plasma α-tocopherol content in ALC compared to ACT in 75 and 100 mg/kg of diet in week 3 obviously shows the higher bioavailability of ALC than ACT at the intestinal level during the weaning period. α-Tocopheryl acetate has to be hydrolysed by pancreatic carboxyl ester hydrolase prior to absorption of the α-tocopherol moiety^7,13^. This discrepancy between absorption of ALC and ACT at the higher inclusion levels indicates that hydrolysis of the acetate ester is the limiting factor in absorption of α-tocopherol in mink kits during the weaning period. This difference between plasma α-tocopherol content in ALC compared to ACT still exists at the age of 7 to 10 weeks (week 6 after supplementation) in mink kits fed 100 and 150 mg/kg of diet but is less pronounced, indicating a higher hydrolysis capacity of the α-tocopheryl acetate bond with increasing age from 7 to 10 weeks. This agrees with previous findings of Hedemann et al.^14^ who reported a twofold increase in the activity of carboxyl ester hydrolase in the pancreatic tissue in relation to the metabolic body weight in mink kits from 5 to 8 weeks of age.

The natural form of α-tocopherol (ALC and ACT) results in a higher plasma α-tocopherol content than the synthetic (SYN) form, which could be due to pre-systemic difference in absorption of the natural form compared to the synthetic form because of carboxyl esterase stereoselectivity of bile salt^15,16^ and post-systemic biodiscrimination against synthetic stereoisomers in SYN. These results agreed with findings of Hymøller et al.^7^.

The linear increase in RRR-α-tocopherol with increasing dose indicates the fact that RRR-α-tocopherol is absorbed at a constant fractional rate. Similarly, Traber et al.^17^ reported a linear relationship between area under curve in a human study with increasing dose of 15, 75 and 150 mg of RRR-α-tocopheryl acetate labelled with deuterium. Although there is a steady influx of α-tocopherol from the diets, the linearity in RRR-α-tocopherol content with increasing doses was still surprising because the enzymes involved in regulation of α-tocopherol are saturable, and a curve linear response was expected^6^. However, most probably the highest α-tocopherol level fed to the mink kits was within normal physiological range and may have been below the saturation level as previously reported in lambs^18^.

Regression analysis of the RRR-α-tocopherol content showed that the RRR-α-tocopherol content cannot be constant in all tissues at all times and is varied based on duration of administration. In agreement with our findings, Weiser et al.^8^ reported that the α-tocopherol concentration of plasma, brain, liver and adipose tissue was time dependent and increased from day 8 to day 115 after supplementation. Most probably, in the present study, continuous intake of α-tocopherol from diets resulted in enrichment of RRR-α-tocopherol during administration from week 3 to 6. In agreement with our findings, it has been reported that the bioavailability of RRR-α-tocopherol and *all-rac*-α-tocopherol is different in α-tocopherol-deficient humans^19,20^ and in healthy non-deficient humans^21^, which obviously proved the influence of a previous RRR-α-tocopherol status on the RRR-α-tocopherol level. Different trends in circulation and accumulation of RRR-α-tocopherol in weeks 3 and 6 were mirrored in different slopes and intercepts in plasma and tissues. In addition, the different slopes and intercepts show that uptake of RRR-α-tocopherol in tissues depends on specific tissue requirements^22^.

Feeding *all-rac*-α-tocopheryl acetate resulted in an enrichment of 2R stereoisomers and a corresponding decrease of 2S-α-tocopherol stereoisomers. Generally, the stereoisomer profile in plasma and tissues of mink kits fed SYN showed a similar pattern with higher content of R configuration stereoisomers, especially RRR-α-tocopherol^7^. In addition, plasma and tissues contained higher percentages of the RRS-α-tocopherol than of the other synthetic 2R forms. A high amount of α-tocopherol stereoisomers with R configuration, especially RRR form, shows that the configuration at C-2 of the α-tocopherol molecule is of major importance with regard to circulation, uptake and storage of α-tocopherol stereoisomers, which has been proved^6,9^. The present findings showed that discrimination between synthetic 2R configuration took place in the following order: RRS > RSS > RSR. In contrast to rats^15^, the chiral centres of the phytyl side chain have greater influence on the distribution of different synthetic 2R-α-tocopherol stereoisomers. The relative bioavailability of RRR-α-tocopherol of SYN was the same in plasma, heart and lungs, but different from liver, where almost no biodiscrimination occurred.

Due to preferential accumulation of R configuration and discrimination against 2S-α-tocopherol in plasma and tissues of mink kits fed SYN, more than 90% of α-tocopherol stereoisomers were present in a 2R configuration. While in liver, the proportion of 2S-α-tocopherol was more than 46% α-tocopherol stereoisomers. The higher proportion of 2S configuration in liver is due to the key role of liver in elimination of 2S-α-tocopherol stereoisomers^9^. In addition, the high proportion of 2S-α-tocopherol in the liver compared to plasma, heart and lungs may be related to the pathways of uptake and transport of α-tocopherol via α-tocopherol binding proteins, which has been shown by Traber et al.^23^ based on liver perfusion studies with d6-RRR-α-tocopheryl acetate and d3-SRR-α-tocopheryl acetate.

## Conclusions

These findings demonstrated the limitation in hydrolysis of ester bond in α-tocopheryl acetate in mink kits around weaning time, especially at the age of 4–7 weeks, and considerable attention has to be paid to the source of α-tocopherol according to the age of mink kits. These results showed that in both weeks 3 and 6 after feeding, there was a higher response in plasma RRR-α-tocopherol of ALC than both ACT and SYN in 75, 100 and 150 mg/kg of diet. In addition, results showed the considerably higher α-tocopherol content in plasma and tissues of mink kits fed ALC compared to ACT. The distinct response in α-tocopherol of plasma and tissues implied that different sources, doses and duration of administration cannot produce equivalent effects, and response depends on dosage and duration of supplementation.

## Materials and methods

This study was carried out according to the guidelines of the Danish Ministry (Justice Law No. 474; 15 May 2014) concerning experiments with animals and care of animals used for experimental purposes under the approval of the Danish Veterinary and Food Administration. The studies were carried out at the Copenhagen Fur Research Centre in Holstebro, Denmark. In addition, study was carried out in compliance with the ARRIVE guidelines**.**

### Animals, housing and feeding

In the present study, 168 brown mink kits were used. In order to find the baseline of α-tocopherol, 12 mink kits were slaughtered in the beginning of the experiment. Then 156 mink kits (12 replicates per treatment group) were randomly assigned to different treatment groups: no added α-tocopherol in the feed (0 dose), RRR-α-tocopherol (ALC), RRR-α-tocopheryl acetate (ACT) or *all-rac*-α-tocopheryl acetate (SYN). At the beginning of the experiment (week 0), the mink kits were 4 weeks of age and nursed by their mother. At 4 weeks of age, the mink kits began to eat solid feed, and at 7 weeks of age (week 3) they were weaned. The mothers and kits were housed in wire-netting rearing cages (L × W × H: 90 × 30 × 45 cm) with wooden nest boxes attached (L × W × H: 24 × 33 × 33 cm). Straw was used for bedding of nests. At weaning, the mother was removed from the litter. All animal facilities were sheltered under permanent outdoor sheds. The animals were fed a standard Danish wet mink feed ration prepared prior to the experiment. Diet composition is presented in Table 4. The animals were fed once a day and had free access to tap water by using an automatic system. Basal diets were mixed, and α-tocopherol supplements were added according to the experimental design and subsequently stored at − 18 °C in 5-kg plastic bags. The necessary number of bags of mixed rations were thawed daily according to intake and fed to the respective treatment groups. The mink kits were fed once a day and had free access to water. α-Tocopherol as RRR-α-tocopherol, RRR-α-tocopheryl acetate or *all-rac*-α-tocopheryl acetate was kindly provided by Provia, Videbaek, Denmark.

**Table 4:** Composition of basal diets (as fed, %).

Ingredients	Diet composition
Fish offal	25
Industrial fish	32
Poultry offal	20
Fish silage	2
Barley and wheat	8.7
Blood meal	3.2
Maize gluten	3
Potato waste	3
Salt	0.39
Vitamin B mix^a^	0.2
Water	2.6
**Nutrient composition**	
Energy (kJ/kg)	6500
Crude protein (%)	29.3
Crude fat (%)	26
Energy distribution^b^	45:40:15

^a^Vitral B-mink super (Agro Korn A/S). B1, 3000 mg/kg; B2, 6000 mg/kg; B6, 5000 mg/kg; folic acid 1000 mg/kg; B12, 20 mg/kg; D-pantothenic acid, 15,000 mg/kg; biotin, 200 mg/kg; choline chloride, 100,000 mg/kg.

^b^Protein:fat:carbohydrates ratio.

### Treatments and study design

The study was carried out as a 6-week dose–response experiment with four different doses in the feed for each RRR-α-tocopherol, RRR-α-tocopheryl acetate or *all-rac*-α-tocopheryl acetate. Moreover, an un-supplemented control group (Control, 0 dose) was included. All diets supplemented with α-tocopherol contained one of the following four doses: 50, 75, 100 or 150 mg/kg of diets. The analysed content of α-tocopherol and α-tocopherol stereoisomers of the diets is shown in Table 1.

Blood samples were taken by heart puncture after euthanising mink kits at the age of 4 weeks (beginning of experiment), 7 weeks (3 weeks after administration of α-tocopherol) and 10 weeks (6 weeks after administration of α-tocopherol). To harvest the plasma, blood samples was centrifuged at 5000 U/min for 15 min. In order to determine the baseline of α-tocopherol stereoisomers in plasma and tissues, additional 12 mink kits were slaughtered at the beginning of the experiment (week 0). In each treatment group, six mink kits were slaughtered 3 weeks (week 3) after starting the experiment, and the last six were slaughtered 6 weeks (week 6) after starting the experiment. Mink kits were fasted for 12 h and subsequently euthanised in CO_2_. The mink kits were opened, and the whole liver, heart and lungs were taken out. The blood and tissue samples were stored at − 18 °C until analysis.

### Fat soluble vitamin analysis

The α-tocopherol concentration in ration, plasma, liver, heart and lungs was analysed by HPLC after saponification and extraction into heptane^15^. Briefly, ration (1.000 g) and plasma (1.00 mL) were diluted with 2.00 mL of ethanol (96% v/v), 0.50 mL of methanol (100%), 1.00 mL of ascorbic acid (20% w/v), 0.30 mL of KOH-water (1:1, w/v) and 0.70 ml of water. Samples were saponified at 80 °C for 20 min. and cooled in the dark. Tissue samples were homogenized in twice the amount of ethanol by an Ultra-Turrax homogenizer (IKA–Werke GmbH, Staufen, Germany) while being kept on ice. Tocopherol was extracted into two volumes of 5.00 mL of heptane, and 100 μL of the combined heptane phase were injected into the HPLC. Aliquots of the homogenates of 167 mg of liver, 200 mg of heart and 200 mg of lung were extracted and saponified with the same procedure as plasma. All solvents used were of HPLC quality. A Perkin-Elmer HS-5-Silica column (4.0 × 125 mm; Perkin-Elmer GmbH, D-7770 Überlingen, Germany) was used for tocopherol determination. The mobile phase was heptane containing 2-propanol (3.0 mL/L), and degassed with helium. The flow rate was 3.0 mL/min. The obtained peak areas and retention time were compared with Merck (D-6100 Darmstadt, Germany) external standards for identification and quantification of the tocopherol. Fluorescence detection was performed with an excitation wavelength of 290 nm and an emission wavelength of 327 nm.

α-Tocopherol stereoisomers were analysed by HPLC. The remaining heptane extract was evaporated to exact dryness under a stream of nitrogen. Then the α-tocopherol was derivatised to its methyl ether according to the method described by Jensen et al.^15^. The methyl ether derivative was extracted with 1.00 mL of heptane, of which 100 µL were injected into the HPLC. Chromatographic separation was achieved on a Chiralcel OD-H column (250 × 0.46 mm, 5 µm particle size), cellulose tris (3,5-dimethylphenylcarbamate) from Daicel Chemical industries, Ltd. (Tokyo, 100–6077, Japan) with heptane as eluent. This method allowed the separation of the eight stereoisomers of α-tocopherol into five peaks: the first peak containing the SRR, SSR, SRS and SSS stereoisomers; the second peak containing the RSS; the third peak containing RRS; the fourth peak containing RRR and the fifth peak containing RSR.

Plasma concentration of 25-OH-D3 and retinol was determined as described by Hymøller and Jensen^24^ and Jensen et al.^25^, respectively. Briefly, after precipitation of proteins in ethanol and methanol, saponification in potassium hydroxide, samples were dried using N_2_, and the residues were re-dissolved in 85% methanol. Separation and quantification were carried out by reverse-phase gradient HPLC and UV-detection at 265 nm, using 1α-hydroxycholecalciferol (Sigma-Aldrich) as the internal standard.

### Calculation and statistical analysis

Tocopherols, α-tocopherol stereoisomers, retinol and 25-OH-D3 were analysed statistically using the PROC MIXED in SAS/STAT® software, Version 9.4 for Windows (Copyright 2013, SAS Institute Inc., Cary, NC, USA). The following normal linear model was used:

$$ {\text{Y}}_{{{\text{ifjk}}}} \, = \,{\text{S}}_{{\text{i}}} \, + \,{\text{D}}_{{\text{f}}} \, + \,{\text{W}}_{{\text{j}}} \, + \,{\text{S}}\, \times \,{\text{D}}_{{{\text{if}}}} \, + \,{\text{S}}\, \times \,{\text{W}}_{{{\text{ij}}}} \, + \,{\text{D}}\, \times \,{\text{W}}_{{{\text{fj}}}} \, + \,{\text{S}}\, \times \,{\text{D}}\, \times \,{\text{W}}_{{{\text{if}}j}} \, + \,\upvarepsilon _{{{\text{ifjk}}}} $$

where Y is the individual dependent variable, S the effect of α-tocopherol source (i = RRR-α-tocopherol, RRR-α-tocopheryl acetate or *all-rac*-α-tocopheryl acetate), D the effect of dose (f = 0, 50, 75, 100 and 150 mg/kg of diets), W the effect of week (j = week 3 after supplementation and week 6 after supplementation), S × D the interaction between source and dose, S × W the interaction between source and week, D × W the interaction between dose and week, and S × D × W the three-way interaction for source, dose and week and k = 1, 2, …, N_ifj_ denote replicates within each combination with N_ifj_ = 6 for the 24 combinations with dose different from zero. The experimental design only included one common control group (0 dose) of 12 mink kits with six mink kits used for week 3 assessments and the remaining six for week 6. We decided to randomize these 12 mink kits to the three sources balancing both on source and week of assessment, i.e., N_i0j_ = 2 for each i and j. Finally, the residual errors ε_ifjk_ were assumed to be independent and N(0,σ^2^) distributed. For dose–response analysis, the effect of dose was changed to a regression in dose instead of the categorical variable above, i.e., βD_ifjk_ instead of D_f_ and excluding ‘dose’ from the CLASS statement of PROC MIXED.

For 50, 75, 100 and 150 mg/kg in SYN, relative bioavailability of RRR-tocopherol in plasma and tissues was calculated as percentage of a given RRR-tocopherol in plasma and tissues divided by percentage of the RRR-tocopherol in the diet. Relative bioavailability of RRR-tocopherol was analysed statistically using the PROC MIXED. The following normal linear model was used:

$$ {\text{Y}}_{{{\text{ifjk}}}} = {\text{ T}}_{{\text{i}}} + {\text{ D}}_{{\text{f}}} + {\text{W}}_{{\text{j}}} + {\text{ T}} \times {\text{D}}_{{{\text{if}}}} + {\text{ T}} \times {\text{W}}_{{{\text{ij}}}} + {\text{ D}} \times {\text{W}}_{{{\text{fj}}}} + {\text{ T}} \times {\text{D}}\, \times \,{\text{W}}_{{{\text{ifj}}}} \, + \,\varepsilon _{{{\text{ifjk}}}} $$

where Y is the individual dependent variable, T the effect of tissue (i = plasma, liver, heart and lungs), D the effect of dose (f = 50, 75, 100 and 150 mg/kg of diets), W the effect of week (j = week 3 after supplementation and week 6 after supplementation), T × D the interaction between tissue and dose, T × W the interaction between tissue and week, D × W the interaction between dose and week, and T × D × W the three-way interaction for tissue, dose and week. All presented *P*-values are from type-2 tests. The values presented in text, Tables and Figures are LS-means unless otherwise noted.
